# A Novel Species of *Penicillium* With Inhibitory Effects Against *Pyricularia oryzae* and Fungal Pathogens Inducing Citrus Diseases

**DOI:** 10.3389/fcimb.2020.604504

**Published:** 2021-02-10

**Authors:** Li-Juan Liang, Rajesh Jeewon, Pem Dhandevi, Siva Sundara Kumar Durairajan, Hongye Li, Fu-Cheng Lin, Hong-Kai Wang

**Affiliations:** ^1^ State Key Laboratory of Rice Biology, Institute of Biotechnology, Zhejiang University, Hangzhou, China; ^2^ Department of Health Sciences, Faculty of Medicine and Health Sciences, University of Mauritius, Reduit, Mauritius; ^3^ Center of Excellence in Fungal Research, Mae Fah Luang University, Chiang Rai, Thailand; ^4^ Division of Mycobiology & Neurodegenerative Disease Research, Department of Microbiology, School of Life Sciences, Central University of Tamil Nadu, Thiruvarur, India

**Keywords:** *Canescentia*, antifungal activity, *Pyricularia oryzae*, citrus diseases, taxonomy, multigene phylogeny, plant pathology

## Abstract

A novel species of *Penicillium*, proposed as *P*. *linzhiense* sp.nov was isolated from soil collected in Linzhi Town, Linzhi County, Tibet Autonomous Region, China. DNA sequence analyses from eight different gene regions indicate that the isolate represents a novel species and most closely related to *P*. *janczewskii*. The phylogenetic analysis based on a concatenated dataset of three genes, ITS, *CaM*, and *BenA*, also confirmed the placement of the novel species within the *Canescentia* section of the genus *Penicillium*. Differences in morphology among similar species are detailed and single gene phylogenies based on ITS, *CaM* and *BenA* genes as well as a multi-loci gene phylogeny are presented. Cultural studies were performed to study inhibitory activities on plant pathogens. The results reveal a notable antifungal activity against *Pyricularia oryzae* causing rice blast with an inhibition rate up to 77%, while for other three citrus pathogens, *Diaporthe citri*, *Phyllosticta citrichinaensis*, and *Colletotrichum gloeosporioides*, inhibition rate was 40, 50, and 55% respectively. No noticeable effects were observed for *Fusarium graminearum*, *Botryosphaeria kuwatsukai*, and *Rhizoctonia solani*. Interestingly, unlike other reported members of *Canescentia*, *P*. *linzhiense* showed no antagonistic effect on root rotting fungi. The new taxon isolated here has the potential to be used as a biocontrol agent especially for economically important phytopathogens or emerging pathogens on diseases occurring on citrus or rice.

## Introduction

Orange is widely planted and consumed, but its production is threatened by a variety of pathogens during production, resulting in huge economic loss every year. To reduce the loss caused by pathogens, a large amount of pesticides have been applied to protect citrus from plant pathogens. Usage of these pesticides is harmful to the environment and human health (Nicolopoulou-Stamati et al., 2016; Bisht et al., 2019). For example, mancozeb, an effective fungicide applied widely to inhibit *D*. *citri* on citrus in China (Chen et al., 2010; Jiang et al., 2012; Liu et al., 2018), is carcinogenic, teratogenic, and mutagenic after degrading to ethylene thiourea (ETU). Besides, with increasing pesticide abuse, pathogens are easily subjected to resistance to pesticides, leading to failure of disease control. Therefore, it is important to screen beneficial fungi for potential biological control leading to the safe production of citrus.

Some fungi are considered as biocontrol agents for inhibiting plant pathogens, but very few are associated with members of the genus *Penicillium*. Studies have reported that conidia or culture filtrate of *P*. *janczewskii* can induce systemic resistance of melon and tobacco to inhibit *Rhizoctonia solani* (Madi and Katan, 1998; Nicoletti et al., 2007), and one of its main secondary metabolites, pseurotin A, reveals moderate inhibition against *Erwinia carotovora* and *Pseudomonas syringae* (Schmeda-Hirschmann et al., 2008).

*Penicillium* is much more ubiquitous in the environment than other species of fungi and they also exist as endophytes inside plant tissues (Rashmi et al., 2019). Due to a paucity of morphological characteristics, overlap of morphs among different species and largely similar cultural characteristics, traditional morphological classification somehow been somehow unreliable to delineate species in this genus (Houbraken et al., 2014).With the development of modern molecular based phylogeny, the concept of multi-loci sequence typing (MLST) for phylogenetic species was proposed in the classification of *Penicillium*, thus making identification of strains more precise (Rakeman et al., 2002). MLST is a typing method initially used for distinguishing bacterial mutations by directly determining the nucleotide sequences of multiple housekeeping genes that are highly conservative and usually stably expressed in all cells. It was first applied by Maiden et al. (1998) in typing of the bacterial pathogen (*Neisseria meningitidis*), gradually implemented to other bacteria and fungi predominantly in epidemiology and taxonomy. Based on multiple gene locus sequence information to identify species, Visagie et al. (2014a) came up with a short standardized gene region namely DNA barcoding which was used in the identification of *Penicillium*. Up to now, there are DNA barcodes for more than 370 species accepted for *Penicillium*. Currently, the internal transcribed spacer rDNA regions (ITS) is widely sequenced as an official DNA barcode when discriminating species for fungi (Schoch et al., 2012; Lücking et al., 2020). However, ITS is not robust enough to identify species in *Penicillium* and alternative barcodes are needed to assist proper identification (Skouboe et al., 1999; Seifert et al., 2007; Visagie et al., 2014a; Nilsson et al., 2018; Tekpinar and Kalmer, 2019). The β-tubulin gene (*BenA*), the calmodulin gene (*CaM*), and the RNA polymerase II second largest subunit gene (*RPB2*) combined with ITS have been successfully employed in species-level identification of *Penicillium* (Houbraken et al., 2014; Visagie et al., 2016a; Visagie et al., 2016b; Wang et al., 2017; Diao et al., 2019). They, however, have certain limitations sometimes, such as difficulties in amplifying specific gene regions and sequence analyses due to ambiguously aligned sites and these lead to problems in resolving species concepts (Giraud et al., 2010; Houbraken et al., 2012; Chen et al., 2013; Wang and Wang, 2013; Visagie et al., 2014a; Visagie et al., 2016b). In this study, multi gene phylogenetic analyses were performed based on ITS, *BenA*, and *CaM* following the recommendations of Visagie et al. (2016b).

During the screening of fungi for potential biocontrol agents on *Citrus*, a fungal strain, Z863, was isolated from soil by selective medium dilution plate method (Houbraken and Samson, 2011). Morphological examinations and DNA sequence analyses reveals that Z863 is a new species belonging to *Penicillium* sect. *Canescentia*. Members of section *Canescentia* are soil inhabitants and there exist several studies demonstrating their potential antifungal activity predominantly related to the inhibition of soil-borne pathogens (Madi and Katan, 1998; Nicoletti et al., 2007; Schmeda-Hirschmann et al., 2008; Houbraken and Samson, 2011; Urooj et al., 2018). The aim of this paper is to introduce this taxon collected from China as a new species based on morphology supported by phylogenetic analyses of a combined dataset from ITS, *BenA*, and *CaM* genetic data. In addition, we also report results based on its potential inhibitory activities on plant pathogens.

## Materials and Methods

### Sampling and Isolations

Soil samples were collected in Linzhi Town, Linzhi County, Tibet Autonomous Region (29.60146 N, 94.41736 E), China. The fungal samples were separated based on selective medium dilution plate method, in which 10 g soil to 90 ml distilled water, were shaken for 10 min at 120 rpm and diluted twice to 10^−1^ and 10^−2^ of the original concentration. Potato dextrose agar (PDA) as an isolation medium was prepared with 200 g potato, 20 g glucose, 18 g agar, 0.3 g chloramphenicol in 1,000 ml ddH_2_O and sterilized at 121°C for 30 min. Three concentrations of diluent of 0.1 ml were separately pipetted into polystyrene Petri dish with 15 ml coagulated PDA and then the sterile coater was used to homogenize the diluent with three replicates for each concentration. After cultured at 25°C for 3 d, colonies were observed and mycelia (through hyphal typing) were transferred to a new PDA plate and once colonies grow up to 3 cm, they were transferred to new plates again.

### Morphological Identification

Macromorphological characters were checked from Czapek’s agar (CZ), Czapek yeast autolysate agar (CYA), malt extract agar (MEA), and yeast extract sucrose agar (YES) media. The medium’s preparation, strain’s inoculation manner, and incubation conditions were performed following the protocols of Visagie et al. (2014b).

After incubation at 25°C for 7 d, plates were checked, observed and colony’s morphology was recorded. The descriptions of color are based on NBS ISCC color name notation. Afterward, 60% lactic acid was used as a floating agent for making slides, and mycelia and conidia were examined under the microscope. Macromorphological and micromorphological details were also examined and recorded by EOS 600D Camera (Canon, China, Beijing) and Leica Microscope DM750 (Leica, China, Shanghai) with an ICC50 Camera and arrangement of photos was done in Adobe Photoshop CC 2018.

### DNA Extraction and PCR

DNA of the samples was extracted by Rapid Fungi Genomic DNA Isolation Kit (Sangon Biotech, Shanghai, China) as the manufacturer’s instructions.

PCR amplifications of the ITS, *BenA*, *CaM*, *RPB2*, translation elongation factor 1-α (*TEF*) regions, the large subunit (LSU) and the small subunit (SSU) of ribosomal DNA gene and tubulin gene were performed with corresponding primers listed in **Table 1**. One amplification reaction consisted of 25 μl Green Taq Mix (Vazyme, Nanjing, China), 2 μl of each primer (10 μM), 2 μl template DNA (30 ng μl^−1^), and 19 μl ddH_2_O in reaction system according to manufacturer’s instructions. PCR reactions were performed by an MG96G PCR instrument (LongGene, Hangzhou, China) with the following procedure: pre-denaturing at 94°C for 2 min; subsequent 35 cycles with denaturing at 94°C for 30 s, annealing at 55°C for 40 s, extending at 72°C for 1 min; the final extension at 72°C for 10 min. After PCR reaction, products were detected by 1% agarose gel electrophoresis. Purification of products was conducted by the DNA gel purification kit (Axygen Biotech, Hangzhou, China).

**Table 1:** Primers for sequence amplification used in the PCR reaction.

Locus	Primer’s name	Sequence (5′→3′)	Reference
**ITS**	ITS1	TCCGTAGGTGAACCTGCGG	(White et al., 1990)
	ITS4	TCCTCCGCTTATTGATATGC	(White et al., 1990)
***BenA***	Bt2a	GGTAACCAAATCGGTGCTGCTTTC	(Glass and Donaldson, 1995)
	Bt2b	ACCCTCAGTGTAGTGACCCTTGGC	(Glass and Donaldson, 1995)
***CaM***	CMD5	CCGAGTACAAGGARGCCTTC	(Hong et al., 2006)
	CMD6	CCGATRGAGGTCATRACGTGG	(Hong et al., 2006)
***RPB2***	5F	GAYGAYMGWGATCAYTTYGG	(Liu et al., 1999)
	7CR	CCCATRGCTTGYTTRCCCAT	(Liu et al., 1999)
***TEF***	CEFF2	GGCTTCAACGTGAAGAACG	(Castlebury et al., 2004)
	CEFR1	CCGTKCAARCCRGAGATGG	(Castlebury et al., 2004)
**LSU**	LR5	ATCCTGAGGGAAACTTC	(White et al., 1990)
	LROR	ACCCGCTGAACTTAAGC	(White et al., 1990)
**SSU**	NS1	GTAGTCATATGCTTGTCTC	(White et al., 1990)
	NS4	CTTCCGTCAATTCCTTTAAG	(White et al., 1990)
***Tubulin***	T12	TAACAACTGCTGGGCCAAGGGTCAC	(O’Donnell and Cigelnik, 1997)
	T22	TCTGGATGTTGTTGGGAATCC	(O’Donnell and Cigelnik, 1997)

### DNA Sequencing and Sequence Analyses

Purified and recovered target DNA fragments were sent to be directly sequenced in an ABI PRISMA377 automatic sequencer (Sangon Biotech, Shanghai, China). Once DNA sequences were obtained, they were verified and then aligned with homologous or similar nucleotide sequences in the GenBank database by BLAST.

Three fragments including ITS, *CaM*, and *BenA* were employed for further comprehensive phylogenetic analyses. Three sets of genetic data (ITS, *CaM*, and *BenA*) were arranged and corrected by BioEdit Sequence Alignment Editor version 7.2.3, and finally a concatenated DNA sequence dataset was analyzed with Maximum Parsimony (MP) and Maximum Likelihood (ML). Phylogenetic analysis for model selections were performed by Modeltest 3.7 based on the lowest Akaike information criterion (AIC) value. The phylogenetic tree was constructed using PAUP 4.0b10 software under different optimality criteria. The stability of tree branches was evaluated by bootstrap with 1,000 replicates. Trees were processed for publication in Adobe Illustrator CC. Taxa used in the phylogenetic analyses are shown in **Table 2**.

**Table 2:** Strains used for phylogenetic analysis.

Species name	Strain number	GenBank accession numbers		
		ITS	*BenA*	*CaM*
*Penicillium canescens*	CBS300.48^T^	AF033493	JX140946	KJ867009
*Penicillium yarmokense*	CBS410.69^T^	KC411757	KJ834502	KJ867013
*Penicillium radiatolobatum*	CBS340.79^T^	KC411745	KP016920	KP016825
*Penicillium murcianum*	CBS161.81^T^	KP016844	KP016924	KP016824
*Penicillium jensenii*	CBS327.59^T^	AY443470	JX140954	AY443490
*Penicillium janczewskii*	CBS221.28^T^	AY157487	KJ834460	KJ867001
*Penicillium dunedinense*	CBS138218^T^	KJ775678	KJ775171	KJ775405
*Penicillium echinatum*	NRRL917^T^	KP016840	KJ866964	KJ867021
*Penicillium griseoazureum*	CBS162.42^T^	KC411679	KP016919	KP016823
*Penicillium nigricans*	CBS354.48^T^	KC411755	KJ866965	KJ867012
*Penicillium corvianum*	DAOMC250517^T^	KT887875	KT887836	KT887797
*Penicillium novaezeelandiae*	CBS137.41^T^	JN617688	KJ834477	KJ866996
*Penicillium coralligerum*	CBS123.65^T^	JN617667	KJ834444	KJ866994
*Penicillium atrovenetum*	CBS241.56^T^	AF033492	JX140944	KJ867004
*Penicillium antarticum*	CBS100492^T^	KJ834503	KJ834432	KP016826
*Penicillium brevicompactum*	CBS257.29^T^	AY484912	AY674437	AY484813
*Penicillium nucicola*	DAOMC250522^T^	KT887860	KT887821	KT887782
*Penicillium janczewskii*	CBS166.81	KC411682	KJ866967	KJ866998
*Penicillium janczewskii*	CBS413.68	KP016838	KJ866969	KJ867014
*Penicillium janczewskii*	CBS279.47	KP016837	KJ866968	KJ867008

### Cultural Studies and Inhibition Activities on Plant Pathogens

Target strain and each tested pathogen (*D*. *citri*, *Ph*. *citrichinaensis*, *C*. *gloeosporioides*, *Py*. *oryzae*, *F*. *graminearum*, *B*. *kuwatsukai*, and *R*. *solani*) were separately inoculated onto the same 9 cm polystyrene Petri dish containing 15 ml PDA, 4 cm between two inoculums. To set up a control group, each pathogen with the same conditions as the test group was individually inoculated in a single medium at the same position in the PDA plate. Then, both groups were cultured at 25°C for 7 d.

A week later, the growth radius was measured (recorded as *R*) of the control group and the inhibition culture group (recorded as *r*). Each replicate was measured three times and the average was calculated. The inhibition rate of pathogen radius (abbreviated as *IR*) was calculated out according to the following formula:

$$IR=\frac{R-r}{R}\times 100\%$$

## Results

### Isolation and Morphology

Twenty isolates with white color colony on PDA medium plate were isolated. The morphology of these isolates was identical when examined under microscope, suggesting that they belong to the same species. One isolate, Z863 was used as representative for further studies. Results of microscopic examination showed that it is characterized by morphologies of the genus *Penicillium*. Based on phenotypic characters, this taxon belongs to the *Canescentia* section of the genus *Penicillium*. The morphological descriptions are provided in the taxonomy section.

### Taxonomy

**Figure 1:**
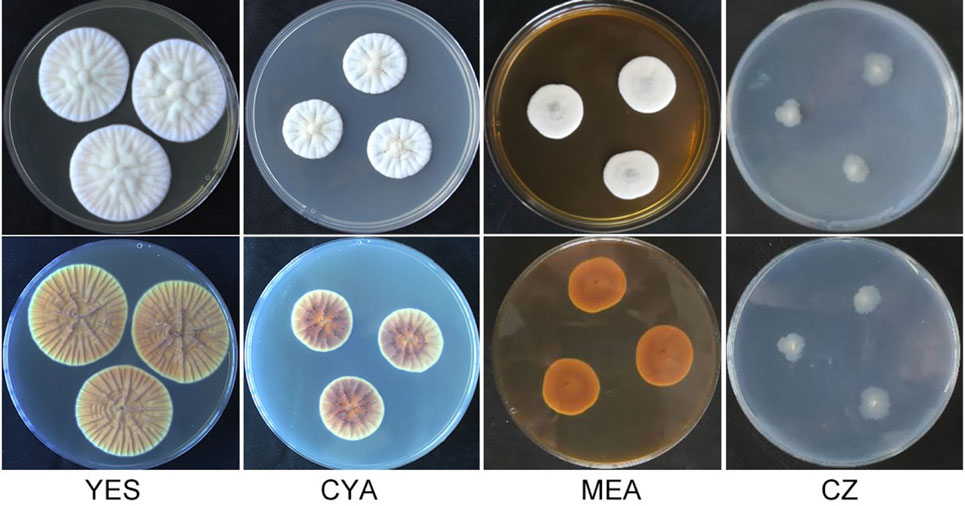
Morphology of *Penicillium linzhiense* after incubation at 25°C on different medium for 7 days. bottom row: reverse plate.

**Figure 2:**
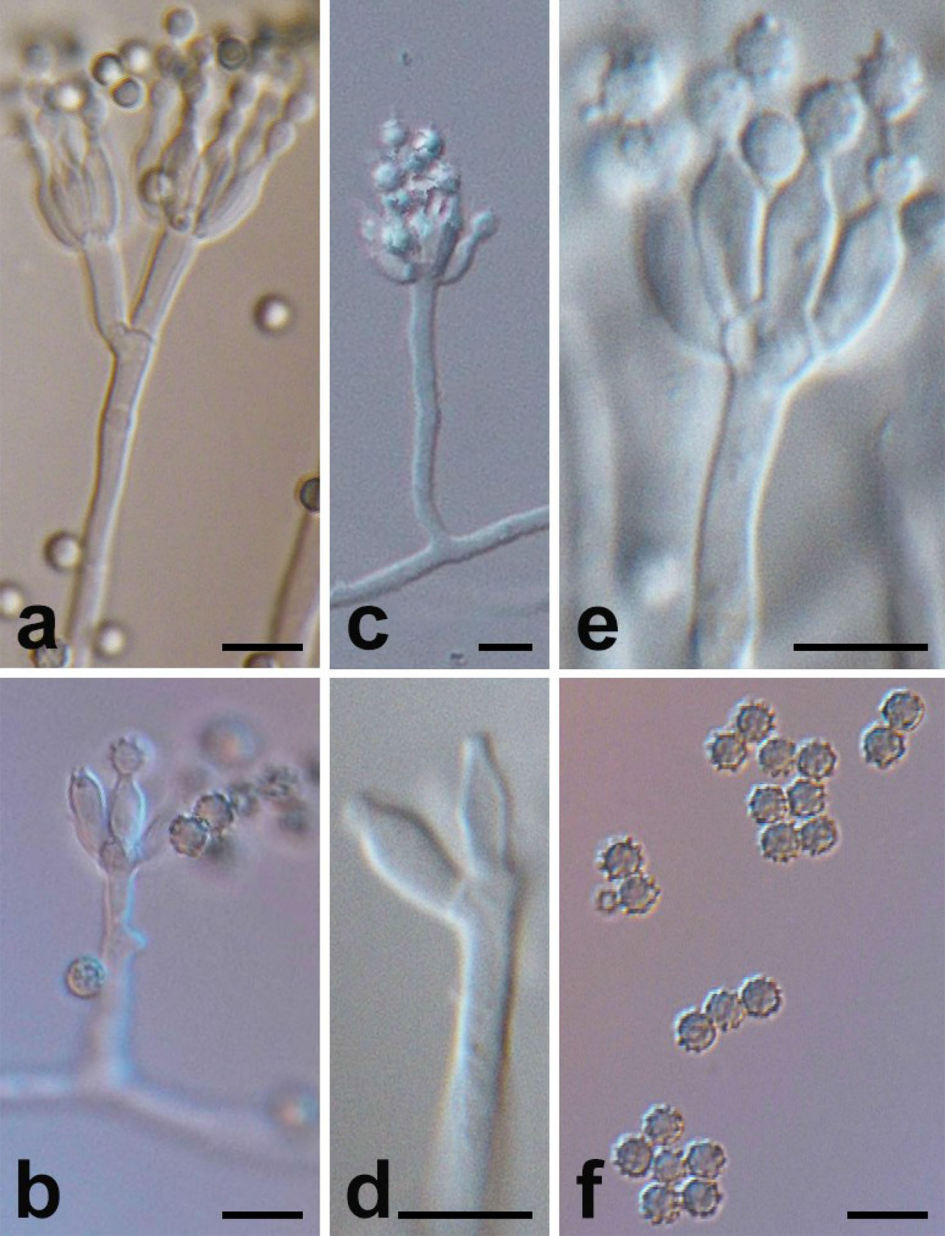
Micromorphology of *P*. *linzhiense*. (Scale bars = 5 μm.) **(A)** Branching status of conidiophore. **(B)** Conidia’s growth status on the bottle stem. **(C)** Conidiophore and bottle stem. **(D)** Morphology of bottle stem. **(E)** Growth manner of bottle stem on base stem. **(F)** Morphology of conidia.

*Penicillium linzhiense* H-K. Wang & R. Jeewon, sp. *nov*. –Mycobank MB#838576; **Figures 1**, **2**.

*In*: subgenus *Penicillium*, section *Canescentia*.

*Barcodes*: ITS-MT461156; *BenA*-MT461157; CaM-MT461162;

*Etymology*. Latin, *linzhiense*, named after Linzhi, China, location where the isolates were collected.

*Type strain examined:* Linzhi Town, Linzhi County, Tibet Autonomous Region (29.60146 N, 94.41736 E), China, 20 Aug. 2016, collected by H-K Wang, CCTCC no: M2019870. Deposited in China typical culture preservation center located in Wuhan University, Wuhan, China.

After incubation at 25°C for 7 d on different medium plates, colonies of *P*. *linzhiense* displayed remarkable differences in morphology (**Figure 1**). Colonies on CYA after 7 d approached 30–32 mm, covered with many radial sulcate, thicker at the center without sulcate; margin entire to somewhat irregular; texture usually flocculent or velvet; mycelia white, moderate yellowish pink (SICC-NBS 29); center white to pinkish white (SICC-NBS 9) with exudate on the surface; soluble pigment absent. Colonies on MEA after 7 d approached 20–30 mm, uneven, navel-like bulge at the center; margin entire to somewhat irregular; mycelia white, texture velutinous; somewhat vivid pink at the center with exudate on the surface; soluble pigment absent. Colonies on CZ after 7 d approached 16–18 mm, low and plane; margin irregular; mycelia (SICC-NBS 184) very pale blue; texture fluffy; exudate and soluble pigment absent. Colonies on YES after 7 d approached 42–44 mm, covered with many radial wrinkles, thicker without radial wrinkles at the center; margin entire; mycelia white, yellowish white (SICC-NBS 92) to pale greenish yellow (SICC-NBS 104); texture mostly flocculent or velvet; exudate and soluble pigment absent.

Microscopic characters were also examined on MEA, conidiophore (**Figure 2C**), 20–100 × 2–2.5 μm, occurred in aerial or dragging hyphae with smooth walls. Broom branches (**Figure 2A**) are predominantly single-whorled, with fewer double-whorls and solitary pedicels with enlarged apices. It grew two to eight or more bottle pedicels (6–8 × 2.0–2.5 μm) per whorl, typically flask-shaped, with short and distinct necks (**Figures 2D–E**). Conidia were spherical or subspherical in shape, 2.6–4.5 μm, markedly spiny and rough (**Figure 2F**), each in a bottle stem or free (**Figure 2B**). Conidial chains were loose, nearly cylindrical, or irregular.

Morphology of conidiophore and conidia of *P*. *linzhiense* are similar to *P*. *janczewskii*. The strain differs from *P*. *janczewskii* in that *P*. *linzhiense* has a light colony on CYA and is grayish-white without becoming grayish-green within 2 weeks; the conidiophore branching pattern is predominantly monoverticillate, with fewer biverticillate, and conidiophore are solitary. However, colonies of *P*. *janczewskii* change from grayish-green to grayish-black on CYA medium; conidiophore branching patterns of *P*. *janczewskii*, *P*. *dunedinense*, *P*. *echinatum*, *P*. *griseoazureum*, *P*. *nigricans* are mainly biverticillate, with terverticillate, or few monoverticillate, with two to four metula per conidiophore. This novel species can be distinguished from *P. corvianum* by the spiny conidia.

### Sequencing and Phylogenetic Analyses

Eight gene fragments of strain Z863 were obtained using PCR according to the primer pairs in **Table 1**. All the sequences were uploaded to GenBank with the following accession numbers (ITS: MT461156; *BenA*: MT461157; LSU: MT461158; SSU: MT461159; *TEF*: MT461160; *RPB2*: MT461161; *CaM*: MT461162; beta-tubulin (*Tub*): MT461163).

**Figure 3:**
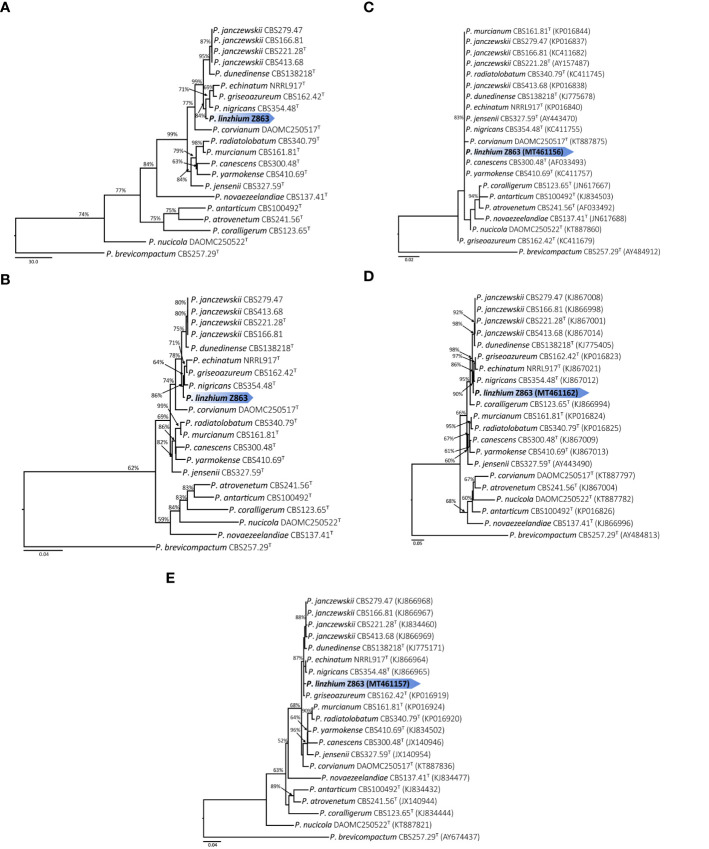
**(A)** Maximum parsimony tree of the combined sequence of ITS, *CaM* and *BenA* of *P*. *linzhiense* (^T^ = ex-type; the scale bar shows the number of substitutions and the values above the nodes represent bootstrap support). The new species is marked by blue block. **(B)** Maximum likelihood tree of the combined sequence of ITS, *CaM*, and *BenA* of *P*. *linzhiense* (^T^ = ex-type; the scale bar shows the number of substitutions and the values above the nodes represent bootstrap support). The new species is marked by blue block. **(C)** Maximum likelihood tree derived from DNA sequence analyses of the ITS, gene region. (The scale bar shows the number of substitutions and the values above the nodes represent bootstrap support, but those support lower than 50% are not showed). **(D)** Maximum likelihood tree derived from DNA sequence analyses of the *CaM* gene region. (The scale bar shows the number of substitutions and the values above the nodes represent bootstrap support, but those support lower than 50% are not showed). **(E)** Maximum likelihood tree derived from DNA sequence analyses of the *BenA*, gene region. (The scale bar shows the number of substitutions and the values above the nodes represent bootstrap support, but those support lower than 50% are not showed).

Phylogenetic analysis (Maximum Likelihood, ML) based on a combined ITS, *CaM*, and *BenA* dataset of 21 taxa (with 1,238 characters and 130 parsimony informative characters) with *P*. *brevicompactum* as outgroup resulted in one tree shown in **Figure 3A** (TL = 513, CI = 0.789, RI = 0.738, RC = 0.583, HI = 0.211). Phylogeny depicts that Z863 is a new species as it constitutes a strongly supported independent lineage basal to *P*. *janczewskii*, *P*. *dunedinense*, *P*. *nigricans*, *P*. *griseoazureum*, and *P*. *echinatum* (**Figure 3A**).

The Maximum Parsimony (MP) phylogenetic tree derived from the combined dataset shown in **Figure 3B** is based on 1,238 characters with best model GTR+G+I. The phylogenetic position of Z863 is the same in the ML tree. This result also confirmed that Z863 is a new species in *Penicillium* sect. *Canescentia*. During our initial ITS sequence BLAST search in GenBank, the similarity between our strain and *P*. *janczewskii* (MK179261), *P*. *arizonense* (MH492021), *P*. *canescens* (KX359603), *P*. *murcianum* (NR_138358), *P*. *janczewskii* (KP016839) was 100%. This confirms that our species is undoubtedly a *Penicillium* species and belongs to *Penicillium* sect. *Canescentia*. However, one cannot rely on ITS alone for proper identification and establishing new species, especially for taxonomically complex genera (Jeewon and Hyde, 2016). Even our single gene phylogenetic analyses based on ITS alone also shows that the tree is unresolved with weak branch support and the affinities of *P. linzhiense* to *P. corvianum* (KT887875), *P. canescens* (AF033493), and P*. yarmokense* (KC411757) are not clear (**Figure 3C**). However single gene datasets based on *CaM* and *BenA* genes provided better resolution (**Figures 3D, E**).

### Inhibition of *Penicillium linzhiense* on Plant Pathogens

**Figure 4:**
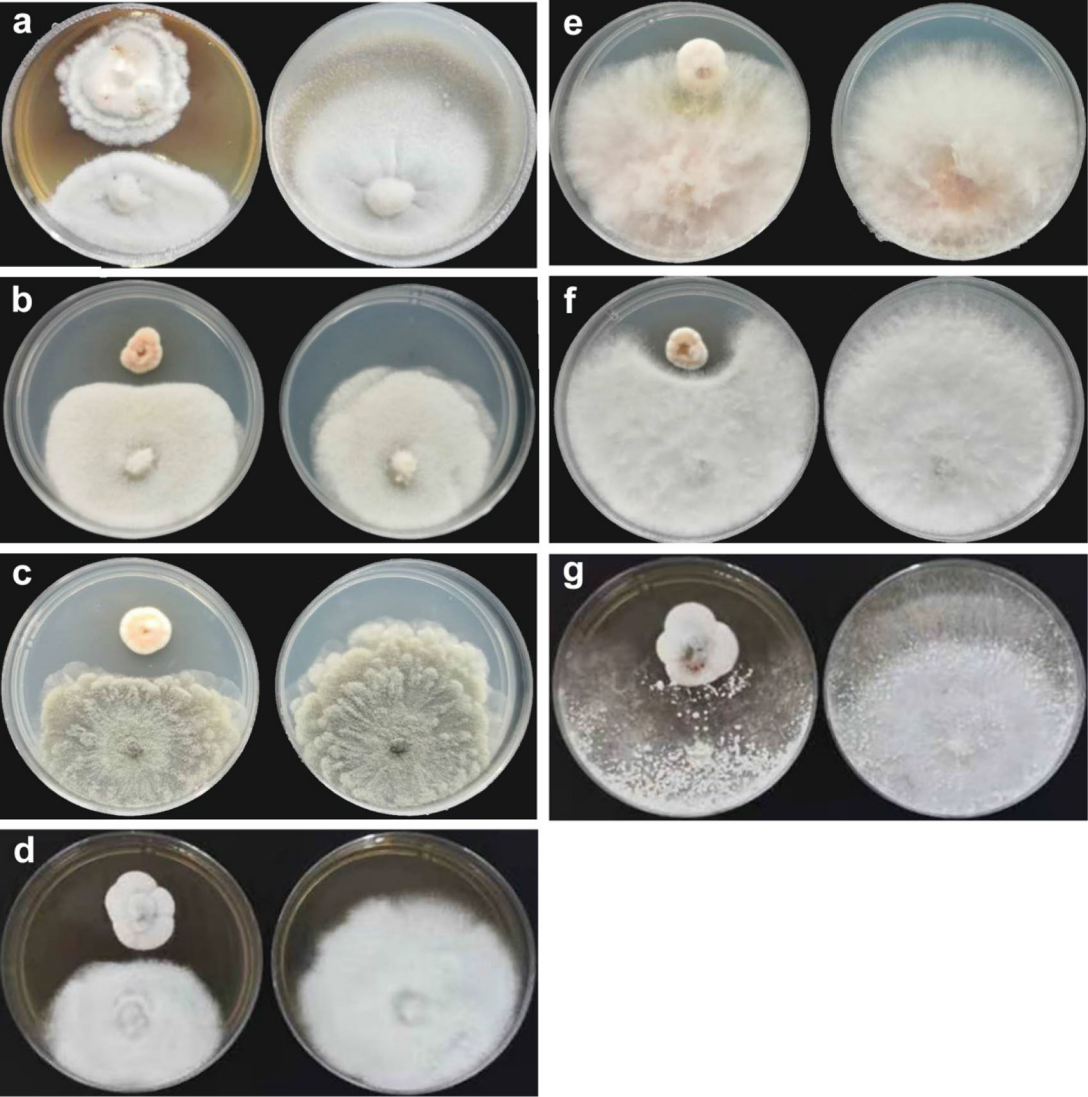
Inhibition of *P*. *linzhiense* on plant pathogens. **(A)** Inhibitory effect of *P*. *linzhiense* on *Pyricularia oryzae* after 14 d; **(B)** Inhibitory effect of *P*. *linzhiense* on *Diaporthe citri* after 7 d; **(C)** Inhibitory effect of *P*. *linzhiense* on *Phyllosticta citrichinaensis* after 7 d; **(D)** Inhibitory effect of *P*. *linzhiense* on *Colletotrichum gloeosporioides* after 7 d; **(E)** Inhibitory effect of *P*. *linzhiense* on *Fusarium graminearum* after 7 d; **(F)** Inhibitory effect of *P*. *linzhiense* on *Botryosphaeria kuwatsukai* after 7 d; **(G)** Inhibitory effect of *P*. *linzhiense* on *Rhizoctonia solani* after 7 d. [**(A–G)** The left one was an inhibition culture group (above: *P*. *linzhiense*; below: the tested pathogen), and the right one was a control group].

**Figure 5:**
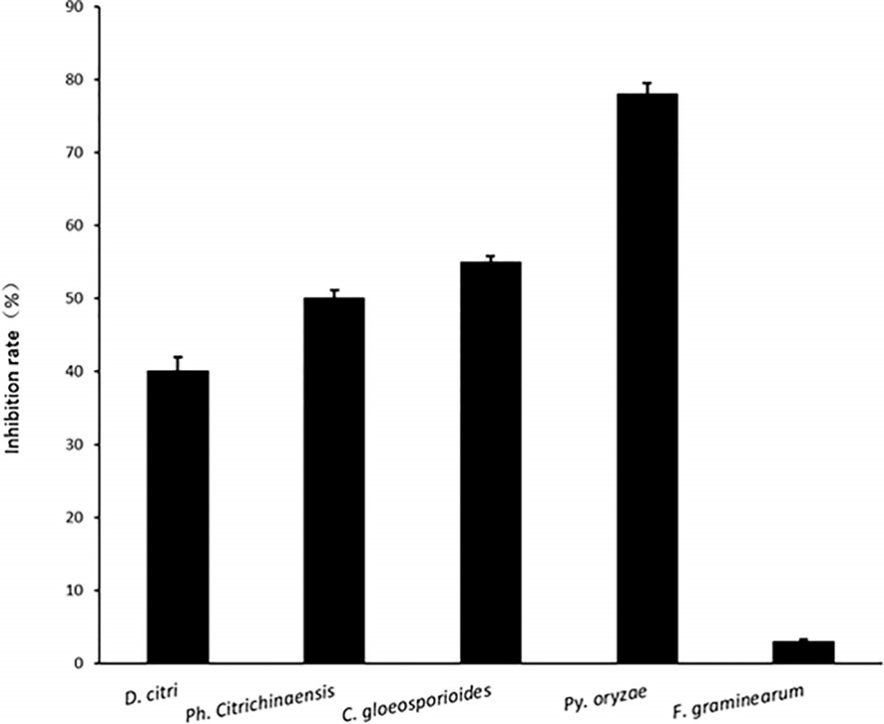
The inhibition rate of pathogen radius (*IR*) of *P*. *linzhiense* against different pathogens in the confrontation culture test.

Comparing inhibition culture group and control group, *P*. *linzhiense* showed inhibitory effects against *Py*. *oryzae* (**Figure 4A**), *D*. *citri* (**Figure 4B**), *Ph*. *citrichinaensis* (**Figure 4C**), and *C*. *gloeosporioides* (**Figure 4D**). However, the strain did not exhibit significant effect on *F*. *graminearum* (**Figure 4E**) and had no inhibition against *B*. *kuwatsukai* (**Figure 4F**) and *R*. *solani* (**Figure 4G**). The inhibition rate of pathogen radius (*IR*) is showed in **Figure 5**.

## Discussion

*Penicillium* is widely distributed in the environment and easily isolated from air and soil. To date many published reports have reported a ubiquitous and high frequency of *Penicillium* in soil samples from different climatic conditions and geographical regions (Grishkan and Nevo, 2004; Sharma et al., 2011; Cruz et al., 2013; Kalashnikova et al., 2016; Cecchi et al., 2019) and these species are associated with important soil function. Despite their important roles, the traditional morphological delineation of species has always been a taxonomic dilemma and currently DNA based sequence data from a combination of different genes especially ITS, *BenA*, and *CaM* should be analyzed for accurate identification. In our study, we isolated a new species, *Penicillium linzhiense* and its morphological details, ability to restrict growth of fungal pathogens and evolutionary relationships are discussed.

Our multigene phylogeny reveals that *P. linzhiense* is close to *P*. *janczewskii*, *P*. *dunedinense*, *P*. *echinata*, *P*. *griseoazureum*, and *P*. *nigricans*. However, *P*. *linzhiense* is distinct from species mentioned above particularly in morphs with distinctly thinner mycelium on CZ medium and with single-whorled broom branches mainly accompanied by few double-whorled one. With respect to *P*. *janczewskii*, *P*. *linzhiense* mainly differs in the color of colony after two weeks’ culture and the broom branches (at the start, there is gray-white and mainly monoverticillate, with fewer biverticillate, with solitary conidiophore, and with time turns grayish green to grayish black and mainly biverticillate, with terverticillate or few monoverticillate, with two to four metula per conidiophore). When the cultural characteristics of *P*. *dunedinense* are compared against *P*. *linzhiense* at 25°C after 7 d, clear differences can be observed. On CYA medium, wrinkles of *P*. *linzhiense* look denser than *P*. *dunedinense*; on MEA, *P*. *dunedinense* is sulcate but *P*. *linzhiense* is not; on YES, *P*. *dunedinense* is grayish orange but *P*. *linzhiense* is white (Visagie et al., 2014b). As for *P*. *echinata*, its conidiophores have diaphragms, conidial chains that are relatively tighter than *P*. *linzhiense* and its broom branches are single-whorled (Matsushima, 1972), but conidiophores of *P*. *linzhiense* have no diaphragms and there exist few double-whorls of broom branches in *P*. * linzhiense*. As is shown in *Manual and Atlas of the Penicillia* (Ramirez and Martinez, 1982), both *P*. *griseoazureum* and *P*. *nigricans* on CYA and on MEA do not have any exudate but *P*. *linzhiense* possesses yellowish brown exudate on the colonies on CYA and MEA.

Phylogenetic analyses of a combined ITS, *CaM*, and *BenA* sequence dataset (**Figures 3A**, **B**) in the study show that *P*. * linzhiense* forms a distinct lineage, basal to *P*. *nigricans* CBS 354.48 with high bootstrap support in ML analysis (84% ML). Based on the recommendations for the establishment of new species proposed by Jeewon and Hyde (2016), we also compared % differences across all genes amplified. Comparison of ITS, *CaM*, and *BenA* nucleotides between *P*. *linzhiense* and *P*. *nigricans* CBS 354.48 reveals 0, 2, and 3 base pair differences, respectively. In the phylogram generated from maximum likelihood analysis based on ITS sequence data, *P*. *linzhiense* was observed to be closely related to *P*. *yarmokense* (CBS 410.69), *P. corvianum* (KT887875), and *P. canescens* (AF033493) (**Figure 3C**) but this relationship is unstable and unresolved. Furthermore, a comparison of DNA sequences of the ITS regions sequences between *P*. *linzhiense* and *P*. *yarmokense* shows 0 (0%) base pair differences. Although ITS barcodes play an important role in the taxonomy of *Penicillium* species, this gene region is not powerful enough to discriminate species due to their low variability (Skouboe et al., 1999; Seifert et al., 2007; Stielow et al., 2015). Upon analysis of the *BenA* sequences data, *P*. *linzhiense* was found to be a sister taxon to *P*. *echinatum* (NRRL917) (**Figure 3E**) and nucleotide comparison reveals 3 (0.8%) base pair differences between these two taxa. Thus, based on the phylogenetic analyses of the concatenated dataset and phenotypic differences and following the guidelines proposed by Jeewon and Hyde (2016), we hereby introduce *P*. *linzhiense* as a new species in the genus *Penicillium*.

In the inhibition studies, as we expected, *P*. *linzhiense* shows a distinct inhibitory effect against three important pathogenic fungi causing citrus diseases. Results also show a stronger inhibition against *Pyricularia oryzae* causing rice blast (**Figure 4**). It is recommended to perform further pathogenicity studies on *P*. *linzhiense*, including field experiments and the effect of metabolites to assess to what extent the latter can be used as a potential biological control agent in integrated disease management strategies. The discovery of *P*. *linzhiense* provides one more possibility to control citrus diseases and rice blast.

Members of *Penicillium* sect. *Canescentia* are well known as soil-borne fungi (Houbraken and Samson, 2011) and some studies pointed out they possess distinct inhibitory effect against root rotting fungi and even able to promote growth of plants (Madi and Katan, 1998; Nicoletti et al., 2007; Schmeda-Hirschmann et al., 2008; Urooj et al., 2018). Interestingly, as a member of *Penicillium* sect. *Canescentia*, *P*. *linzhiense* shows little inhibition against common soil-borne pathogens unlike other reported members mentioned above. Instead, *P*. *linzhiense* reveals its suppression on pathogens triggering plant disease aboveground. As revealed in this study, results demonstrate that *P*. *linzhiense* can be a potential biocontrol agent for *Py*. *oryzae* which causes damage to the leaves, stems, and ears of rice; *D*. *citri* which causes diseases at the tip of trees, new leaves, and fruits of citrus; *Ph*. *citrichinaensis* which is often found associated with fruits of citrus (Baayen et al., 2002); and *C*. *gloeosporioides* which is detrimental to leaves, branches, flowers, fruits, and fruit stalks of citrus.

## Data Availability Statement

The datasets presented in this study can be found in online repositories. The names of the repository/repositories and accession number(s) can be found below: https://www.ncbi.nlm.nih.gov/genbank/, MT461156; https://www.ncbi.nlm.nih.gov/genbank/, MT461157; https://www.ncbi.nlm.nih.gov/genbank/, MT461158; https://www.ncbi.nlm.nih.gov/genbank/, MT461159; https://www.ncbi.nlm.nih.gov/genbank/, MT461160; https://www.ncbi.nlm.nih.gov/genbank/, MT461161; https://www.ncbi.nlm.nih.gov/genbank/, MT461162; https://www.ncbi.nlm.nih.gov/genbank/, MT461163.

## Author Contributions

RJ, HL, F-CL, and H-KW designed the study. LL did the sample collection and laboratory work. LL, RJ, PD, SSKD and H-KW are involved in phylogenetic analyses and initial writing and finalizing drafts. HL, F-CL, and H-KW contributed for the research funds. All authors contributed to the article and approved the submitted version.

## Funding

This research is supported by the Key R & D Program of Zhejiang Province (2019C02022), China, and the National Key R & D Program of China (2017YFD0202004). RJ thanks the University of Mauritius for support and the MRC funded project MRC/RUN/1705.

## Conflict of Interest

The authors declare that the research was conducted in the absence of any commercial or financial relationships that could be construed as a potential conflict of interest.
